# Suppression force-fields and diffuse competition: competition de-escalation is an evolutionarily stable strategy

**DOI:** 10.1098/rsos.230222

**Published:** 2023-08-30

**Authors:** Daniel Z. Atwater

**Affiliations:** Department of Animal & Range Sciences, Montana State University, 103 Animal Biosciences Building, Bozeman, MT 59717, USA

**Keywords:** competition field, competitive response, game theory, mean-field game model, multiplayer competition, suppression field

## Abstract

Competition theory is founded on the premise that individuals benefit from harming their competitors, which helps them secure resources and prevent inhibition by neighbours. When multiple individuals compete, however, competition has complex indirect effects that reverberate through competitive neighbourhoods. The consequences of such ‘diffuse’ competition are poorly understood. For example, competitive effects may dilute as they propagate through a neighbourhood, weakening benefits of neighbour suppression. Another possibility is that competitive effects may rebound on strong competitors, as their inhibitory effects on their neighbours benefit other competitors in the community. Diffuse competition is unintuitive in part because we lack a clear conceptual framework for understanding how individual interactions manifest in communities of multiple competitors. Here, I use mathematical and agent-based models to illustrate that diffuse interactions—as opposed to direct pairwise interactions—are probably the dominant mode of interaction among multiple competitors. Consequently, competitive effects may regularly rebound, incurring fitness costs under certain conditions, especially when kin–kin interactions are common. These models provide a powerful framework for investigating competitive ability and its evolution and produce clear predictions in ecologically realistic scenarios.

## 1. Introduction

Nature is often described as a battleground over meagre resources; either through competition or predation, survival depends on wresting resources from unwilling victims. Yet, natural examples of cooperation (directly benefitting another organism) and even altruism (directly benefitting another organism at a cost to the individual) abound, with major impacts on ecology and evolution [1–3]. To give a few examples, cooperation is crucial in avoiding ‘tragedies of the commons' that occur from over-exploitation of resources or suboptimal performance by non-cooperators (also called ‘cheaters’ or ‘defectors’; [4]), it facilitates the coexistence of competitors [5], increases speciation [6], and promotes the evolution of phenotypic diversity, behavioural complexity and sociality [3,7,8]. In the simplest terms, cooperation can evolve when its benefits outweigh its costs, although this is complicated by processes such as kin interactions, spatial structure and learning [3].

**Figure 1.**
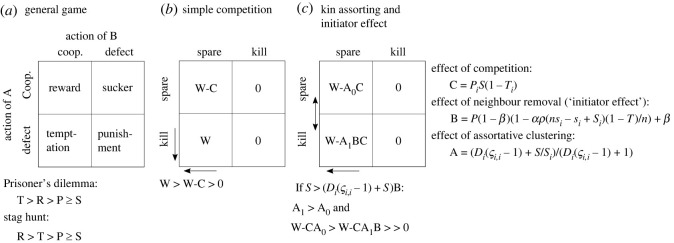
Summary of competition games. The general game (*a*) shows an example payout matrix and payout rankings. In the simple competition game (*b*) defectors kill neighbours. Leaving neighbours alive incurs a competition cost (C) that reduces fitness compared with the maximum (W). In the game with kin assorting and competitor replacement (*c*), agents that kill their competitors might experience relief from competition in the future, as described by the initiator effects term ‘B’. The competitive environment of cooperators versus defectors varies according to the probability of encountering kin, because kin have a known genotype. This is described by term A (subset 0: cooperator, subset 1: defector). Equations for each term are given at right and rankings of the pay-outs is given below. The direction of selection on cooperation or defection is given by vertical arrows. The horizontal arrow shows that agent A would always prefer to survive competition, i.e. by increasing its tolerance.

The evolution of cooperation has been a focus of evolutionary game theory. At the heart of game theory is the ‘pay-off matrix’, which describes the outcome of each combination of strategies used by interacting agents. One well-known example is the Prisoner's Dilemma, in which two agents may cooperate or defect (i.e. forgo cooperation and instead adopt an antagonistic strategy). If both cooperate, both benefit (the reward, R). The greatest pay-out comes to an agent who defects while their partner cooperates (the temptation, T). Conversely, the least pay-out comes to the cooperator in this condition (the sucker, S). When both agents choose to defect, they receive little pay-out (the punishment, P), but this is greater than the pay-out given to a sucker (figure 1*a*). The Hawk-Dove Game is another game that is commonly applied to ecological systems—and it would seem to be relevant where competitive aggression is being explored—but the Prisoner's Dilemma is close mathematically to the models developed here and Hawk-Dove Game is not, so it will not be discussed further.

In the Prisoner's Dilemma the most reliable strategy is to defect because it has the best pay-off no matter which strategy the partner uses. Defection, in the Prisoner's Dilemma, is described as an ‘evolutionarily stable strategy’ (ESS) because it is an ‘evolutionary attractor’ (it tends to evolve from a given starting point) and is ‘invasion-resistant’ (it remains the best strategy once dominant; [9,10]). Evolution therefore drives such a system towards a tragedy of the commons; defection is favoured, the proportion of defectors inevitably increases, and P (both defect) becomes the most common pay-out despite being poor compared with R (both cooperate). Relating this to ecological systems, in which a large pay-out might be interpreted as a boost to growth rate, survival or fecundity, a system with poor pay-outs might have much lower productivity than a system with higher pay-outs. This is unfortunate for the Prisoner's Dilemma, or any other game that favours defection, because evolution inevitably leads to lower community-wide pay-outs and drops in productivity. Game models have been useful in describing real ecological systems, including hunting, root foraging, competition for light, herbivore defence, sex ratios, body size, mate choice, parental care, animal contests, socialization, cancer proliferation and multicellularity [11]. Here, I evaluate how cooperation, in the form of conflict de-escalation, might evolve in populations of direct competitors in the absence of any other trade-offs. This distinction is important, because the traditional paradigm has been to assume that competition inherently drives organisms towards conflicts, and trade-offs are necessary to permit the evolution of cooperation among organisms whose only mode of interaction is competition.

At first glance, interference competition would seem to be a poor candidate for the evolution of cooperation because it might not be obvious why any individual should abstain from harming its competitors, especially if there are no direct costs for doing so and there is a guarantee that competitors are also causing harm. This view of competition centres on a phenomenon I term the ‘initiator effect’, which is the benefit to an individual who pre-emptively suppresses competitors. The initiator effect improves resource availability by weakening or killing competitors and ensures that those competitors cannot exert direct interference effects of their own. However, assortativity of kin, caused by processes such as dispersal limitation or socialization, can help cooperation evolve in social dilemmas because harming others is risky if they might be relatives [12]. Furthermore, multiplayer competition can reduce the benefits of defection through ‘diffuse competition’ among defectors [13,14], meaning that competitive effects are distributed broadly among multiple competitors and initiator effects are diluted.

Diffuse competition has many facets—as explained below—but it can be conceptually thought of as an ‘averaging’ or ‘blending’ of competitive interactions that occur when multiple individuals compete simultaneously in a large arena. Although we tend to view competition in a pairwise lens, a major—or perhaps dominant—form of competition is diffuse. Diffuse competition (DC) has three aspects [15]: Type I: the additive effect of competition, according to the abundance and competitive ability of each species in a competitive neighbourhood [16]; Type II: the cumulative effect of indirect interactions propagating among multiple neighbouring or otherwise interacting competitors [13]; and Type III: the effect of sampling, wherein the average competition experienced by individuals in a population is governed by sampling processes reflecting underlying encounter probabilities with various potential competitors [17]. These aspects of DC all originate as an emergent consequence of multiplayer competition among individuals, and act simultaneously to distribute competitive effects throughout a community, in turn diluting the initiator effect and potentially reducing pay-outs to individuals that suppress direct competitors [14,18–22]. Thus, there is a possibility that kin interactions and diffuse competition could act in tandem to dilute the initiator effect and promote cooperation among competitors. Diffuse competition is an emergent property of multi-competitor systems and operates side-by-side with the individual-based, pairwise interactions that are usually considered by ecologists.

In this paper I develop a series of models to evaluate the possibility of cooperation, in the form of conflict de-escalation, evolving among competitors in the absence of any other ecological trade-offs. Specifically, I investigate how kin assortativity and diffuse competition affect evolution of competitive de-escalation. In ecological terms, defection might involve suppression of competitors through conflict, allelopathy or resource pre-emption, and cooperation—in the form of de-escalation—might involve abstaining from those activities. Furthermore, I allow agents to influence competitive outcomes through variation in their own competitive tolerance. I test predictions of mathematical models with agent-based simulations. My goals with this study are (i) to investigate how kin assortativity and diffuse competition interact to affect evolution of competitive de-escalation, (ii) to explore under what conditions and how often de-escalation might be favoured, (iii) to evaluate how the density and identity of competitors influence the number and nature of ESSs, and (iv) to evaluate equilibrium systems for invasion by other strategies. Ultimately, I aim to demonstrate whether, when and how often cooperation in the form of conflict de-escalation might be an evolutionarily stable competitive strategy even when there is no direct cost of competitive aggression and no other mitigating ecological factors or trade-offs.

A note on terminology: throughout this manuscript I am defining competition as any negative interaction between two individuals, but for simplicity it may be best to think in terms of interference competition, meaning direct suppression (e.g. through attack or allelopathy) of one competitor by another. Resource competition is more complicated because it is an indirect interaction mediated by a resource, which will not be modelled here, although many of the same principles may apply. Also, because competition in these models always has negative effects, cooperation involves conflict de-escalation but never a total loss of competition. As I will show below, even de-escalation is a form of altruism because it comes at the expense of individual survival probability (if not the genotype's inclusive fitness). Fitness is the lifetime reproductive success of an organism, although in the models presented here, variation in fitness is a consequence only of variation in survival, which in turn affects reproductive success.

## 2. The model–game theoretic context

Imagine a scenario in which competitors can kill one another. In game theoretic terms, this is an extreme version of the Prisoner's Dilemma in which defectors kill their partners and two defectors kill one another. A defector whose neighbour cooperates enjoys its maximum fitness, W, because it experiences no competition from its killed neighbour. Throughout the manuscript, I will define ‘cooperators’ and ‘defectors’ in game-theoretic terms (referring to whether an agent chooses how much to harm a partner), although this differs slightly from the ecological definition of cooperation, meaning that an organism acts directly for the benefit of another [6]. The pay-out for mutual cooperators is W – C, where C is the ‘competition term’ describing harmful effects of competition with the surviving neighbour, such as the risk of being killed by that neighbour in the future. For C > 0, it is always advantageous to kill or otherwise suppress competitors, all else being equal (figure 1*b*).

Expressing competition as above implies that there is a possibility of further interaction with the same or other agents. This is called a ‘repeated game’, and it creates a problem for the simple competition model (figure 1*b*), which makes no allowances for continued interaction among agents. If the game is not repeated, then two cooperators are eternally locked into cooperation and C = 0 for the reward case, in which case there is no benefit to defection! If the game is a repeated game, then mutual cooperators and defectors alike might face competition in the future, in which case C needs to be defined for the defector-versus-cooperator case as well as for the mutual cooperation case. To accommodate this, one can introduce a term ‘B’ which describes the proportionate degree of competition felt by defectors as compared with cooperators. I call this the ‘initiator effect’. The initiator effect describes the indirect advantage enjoyed by individuals who suppress their competitors and experience reduced competition in turn. This is one form of diffuse interaction [13,15]. When initiator effects are strong, B ≈ 0, defectors enjoy a considerable advantage. When initiator effects are weak, B ≈ 1, defectors experience little relief from competition, and fitness is negligibly different from that of cooperators. Initiator effects allow interacting genotypes to repeat the game (although the same cannot be said for individuals, which cannot repeat the game once killed).

Initiator effects would seem to prohibit evolutionary de-escalation, by giving suppressors an advantage over de-escalators under all conditions. However, initiator effects could be counteracted if individuals have increased odds of encountering—and harming—relatives. Such kin assortativity generally promotes cooperation in social dilemmas because it increases the risk of defectors harming relatives [12]. I represent kin assortativity with a term ‘A’ which describes the competition felt by a genotype (or kin group) proportionate to the average competition felt by the population. By definition, A varies among genotypes. In a two-agent, non-mixed-strategy example, one can define A_0_ and A_1_, which are the kin-competition terms for competition felt by cooperators and defectors respectively, in which defectors are competitive escalators with strong suppression of competitors, and cooperators are de-escalators with weak suppression of competitors. If kin assortativity is positive, A_1_ > 1 > A_0_, meaning that defectors experience stronger competition than cooperators. This is intuitive; clustered, related defectors will kill one another off, and clustered cooperating relatives will benefit from avoiding interactions with other genotypes which are likely to be stronger suppressors (figure 1*c*). In this more general model, it becomes clear that the evolution of cooperation will depend on the balance between initiator effects and kin-assortativity effects. Cooperation will be favoured when the combined initiator effects and kin-assortativity effects acting on defectors exceed the kin-assortativity effects acting on cooperators,

2.1 $$B>\;\frac{A_{0}}{A_{1}}↔A_{1}B>A_{0}.$$

In other words, for defectors, the benefits of killing neighbours, multiplied by the costs of killing kin, must not exceed the benefits of cooperators sparing kin. What this means in plain terms is that a successful strategy will balance (i) the benefits of cooperating with relatives, (ii) the benefits of killing neighbours, and (iii) the costs of inadvertently killing relatives in the process of killing other neighbours. Fascinatingly, the cost of cooperators sparing unrelated individuals, who may subsequently cause harm to the cooperators, does not need to be considered.

## 3. A suppression-field model of competition

Although an individual may attempt to defect (i.e. by killing a competitor), it is not guaranteed to succeed. It is widely recognized in plant ecology, for example, that competitive ability is the product of two functional traits: the ability to suppress competitors, and the ability to tolerate competition. Thus, the probability of an individual of genotype *i* surviving competition is a function of its own tolerance ability, ${\hat{T}}_{i}$ (0–1; table 1), the average suppression it experiences, ${\hat{s}}_{i}$, (0–1) and its maximum survival probability *P_i_*. The reduction in survival due to competition is term ‘C’, discussed above. We follow Atwater *et al*. [14] in defining this as

3.1 $${\hat{P}}_{i}=P_{i}-C=\;P_{i}-\;P_{i}{\hat{s}}_{i}(1-{\hat{T}}_{i}).$$

Note that in equation (3.1), survival probability is the only aspect of fitness being considered, which in turn leads to changes in reproductive success. In ecological systems, other factors (e.g. fecundity) also certainly play a role, and may trade-off with survival. For the purposes of simplicity, and as these models represent a ‘first-step’, variation in fecundity and other factors are ignored in this study.

**Table 1.** Interpretation of the effects of individual parameters on initiator effects (B), effective survival probability (${\hat{P}}_{i}$), and the tendency for cooperation to evolve. Note that a higher value of B (approaching 1) indicates a weak initiator effect, and a lower value (approaching 0) indicates a stronger effect.

parameter	description	range	effect on B	effect on ${\hat{P}}_{i}$	effect on evolution of cooperation	cooperation is favoured when…	notes
$\bar{P}$	mean pop. survival	0–1	↑ or 0*	↓ or 0*	↓ or 0*	…species are shorter-lived	*0 if β=1
${\bar{s}}_{i}$	mean sup. felt by *i*	0–1	↓ or 0*	↓	↓ or 0*	…individuals feel less suppression	*0 if β=11
$\bar{T}$	mean pop. tol.	0–1	↑ or 0*	↓ or 0*	↑ or 0*	…the species is more competition tolerant	*0 if β=1
β	replacement rate	0–1	↑	↓	↑	…killed competitors are quickly replaced	
α	mean maturity	0–1	↓ or 0*	↓	↓ or 0*	…the population is less mature	*0 if β=1
ρ	mean density	0–1	↓ or 0*	↓	↓ or 0*	…the population is less dense	*0 if β=1
n	neighbourhood size	1–∞	↑ or 0*	↓ or 0*	↑ or 0*	…there are more neighbouring competitors	*0 if β=1
$\varphi_{ii}$	self-encounter rate	0–∞	↑ or ↓*	↑ or ↓*	↓ or ↑*	…kin encounters are common (with exceptions)	*depends upon β and $S_{i}$
$P_{i}$	max. survival of *i*	0–1	0	↑	↓ or 0*	…maximum lifetimes are short	*0 if β=1
$S_{i}$	sup. of *i*	0–1	↓	↑ or ↓*	↓ or ↑*	n/a	*depends upon β and $\varsigma_{i,i}$
$T_{i}$	tol. of *i*	0–1	0	↑	↑ or 0*	…individuals are more competition tolerant	*0 if β=1

In equation (3.1), variables$\;{\hat{T}}_{i}$ and *P_i_* are intrinsic traits of a genotype, but ${\hat{s}}_{i}$, the suppression experienced by individuals of genotype *i* is influenced by the competitive ability of interaction partners and must be calculated. Let us assume that, for any given individual, $\hat{s}$ is the mean age-dependent suppression caused by *n* neighbouring or otherwise interacting competitors (Type I DC, per [15,16]),

3.2 $$\hat{s}={\hat{s}}_{i}=\alpha \rho \overset{k}{\underset{\;j=1}{\sum }}D_{j}S_{j}\;\mathrm{such}\;\mathrm{that}\;\overset{k}{\underset{\;j=1}{\sum }}D_{j}=1.$$

For the typical individual of genotype *i*, the suppression experienced is a function of the suppression of each genotype in the population multiplied by the probability of encountering that genotype, where the encounter probability is the relative density of each genotype (*D_j_*) multiplied by the total relative population density, *ρ* (0–1; table 1 for a list of variables and ranges). For consistency with simulations (discussed in the next section), I also include age effects, *α*, representing the ‘effective maturity’ of the population; a value close to one means that most individuals are mature or nearly mature, and competition is relatively intense. A value close to zero means that age effects are strong, the average individual is not close to maturity, and competition is weakened proportionately.

As defined here, $\hat{s}$ is the same for all *k* genotypes (because other variables are properties of the system and not unique to any given genotype), acting as a uniform field that equally suppresses all genotypes in the population. This formulation represents Type III DC, in which competitive effects are regularized across a population by statistical averaging [17]. This is also called a ‘mean-field’ approximation, which is used in physics, biology, economics and social science to reduce the dimensionality of complex systems with many interacting agents [23]. Analogous to an electromagnetic field created by the action of many individual particles, suppression in this model functions as a field of force affecting survival probability (and, in theory, also including growth and/or fecundity). Critically, in real populations the suppression experienced by any one individual is not equal to$\;\hat{s}$; instead, it is uniquely defined by that individual's set of neighbourhood interactions. However, because that set is sampled from a distribution of possible values, the mean suppression experienced by individuals in the population approaches $\hat{s}$ as population size approaches infinity [17], with variation due to sampling error.

Equation (3.2) assumes that all genotypes experience the same competitive environment, but that may not be the case in nature. For example, kin assortativity, caused by dispersal limitation, clustering of family groups, or kin avoidance, might lead to predictable variation in the competitive environment of kin groups (term A, above) [12,14]. I introduce an ‘association’ parameter, *φ_ij_*, which generalizes equation (3.1) to

3.3 $${\hat{s}}_{i}=\alpha \rho \overset{k}{\underset{\;j=1}{\sum }}\frac{\varphi_{ij}}{D_{j}(\varphi_{ij}-1)+1}D_{j}S_{j},$$

where *φ_i,j_* is an odds ratio comparing odds of genotype *i* encountering genotype *j* versus any other genotype. Assuming that dispersal limitation affects the odds ratio of a genotype encountering itself, *φ_ii_*, but does not bias odds ratios against any other genotypes, *φ_i_*_o_, it follows that

3.4 $${\hat{s}}_{i}=\alpha \rho \frac{D_{i}S_{i}(\varphi_{ii}-1)+\bar{S}}{D_{i}(\varphi_{ii}-1)+1}.$$

As *φ_ii_* increases above 1, *S_i_* becomes an increasingly important component of ${\hat{s}}_{i}$, the correlation between *S_i_* and $\hat{P}$ becomes more negative, and fitness decreases. This model incorporates Type III DC and also allows the competitive environment to vary among genotypes but it does not explicitly include Type I DC, and Type II DC—propagating indirect interactions in a group of neighbouring competitors—is absent. To include such ‘neighbourhood interactions', it is necessary to incorporate terms that represent effects of successful ousting of competitors on ${\hat{s}}_{i}$ and its consequences for not just the individual but the community as a whole. I note that the competitive ‘neighbourhood’ does not need to represent an actual group of neighbours in shared space—it could just as easily refer to a network of individuals that interact in space or time. These models are space-agnostic.

As explained above, the more an individual of genotype *i* harms, kills or weakens a competitor, the more it will reduce the suppression it experiences (${\hat{s}}_{i}$) from those competitors, assuming they are not immediately replaced if killed. To incorporate such ‘initiator effects’, I introduce a term, B, to equation (2.1) (analogous to term B in the game models),

3.5 $${\hat{P}}_{i}=P_{i}-P_{i}{\hat{s}}_{i}B(1-T_{i}),$$

where $B=\bar{P}(1-\beta )(1-(1/n)(n{\hat{s}}_{i}-{\hat{s}}_{i}+\alpha \rho S_{i})(1-\bar{T}))+\beta$.

Term B, which ranges from 0 to 1, describes how neighbour suppression reciprocally ameliorates the competition experienced by an individual in the future—i.e. the initiator effect (see the electronic supplementary material for how this term is derived). The variable *β* describes the replacement rate of killed neighbours, decreasing from one to zero in proportion to recruitment delays, age effects, or incomplete dispersal. A greater replacement rate, *β*, indicates that killed competitors are quickly replaced, which brings B closer to one, reducing benefits of neighbour suppression via neighbourhood effects. Note that equation (3.5) reduces to equation (2.1) for *β* = 1, in which case killed competitors are immediately replaced by equivalent competitors. For simplicity, I have chosen to model *β* as an independent parameter, but more realistically *β* might co-vary with *α* and *ρ* and could possibly be expressed as a function of those. Equation (3.5) contributes Type II DC to the model [13,15], and introduces Type I DC explicitly in the central term containing *n*.

We can conceptualize suppression as a force affecting the survival probability of each individual in the community. The suppression experienced by the whole community could therefore be mapped as a field of force, conceptually similar to a magnetic field. If the force is entirely uniform (i.e. ${\hat{s}}_{i}$ is the same everywhere), all genotypes encounter the same field strength (C; mean-field effect; equation (3.2)). Addition of either initiator effects (B) or kin assortativity (A) modifies the suppression force-field model (hereafter, SFM) to differ among genotypes. Assembling these terms produces the complete SFM explored in this study,

$${\hat{P}}_{i}=ABC$$

Kin-assortativity effect: $A=({\hat{S}}_{i}/\bar{S})=\alpha \rho ((D_{i}S_{i}/\bar{S})(\varphi_{ii}-1)+1/D_{i}(\varphi_{ii}-1)+1)$
Initiator effect: $B=\bar{P}(1-\beta )(1-(1/n)(n{\hat{s}}_{i}-{\hat{s}}_{i}+\alpha \rho S_{i})(1-\bar{T}))+\beta$
Mean-field effect: $C=\;P\bar{S}(1-\hat{T})$
  3.6 $${\hat{P}}_{i}=\;P_{i}-P_{i}\alpha \rho \frac{D_{i}S_{i}(\;\varphi_{ii}-1)+\bar{S}}{D_{i}(\;\varphi_{ii}-1)+1}\left(\bar{P}(1-\beta )\left(1-\frac{\alpha \rho }{n}\left((n-1)\frac{D_{i}S_{i}(\;\varphi_{ii}-1)+\bar{S}}{D_{i}(\;\varphi_{ii}-1)+1}+S_{i}\right)(1-\bar{T})\right)+\beta \right)(1-T_{i}).$$

## 4. Iterating suppression-field models

I simulated SFMs using genotypes that varied in every possible combination of 4, 13 or 101 values of *T* and *S* ranging from 0.2 to 0.8 (for a total of 16, 169 or 10 201 genotypes). Genotypes started at equal densities. To simulate passage of time, I estimated the survival probability $\hat{P_{i}}$ of each genotype. I then scaled *D_i_* to $\hat{P_{i}}$ and ran the model again. These two steps were repeated for 200 time steps, because this was sufficient to allow strong responses to selection in the simulation models. Reproduction was clonal, and the model progressed in discrete time intervals. I varied kin assortativity *φ_ii_* = 0, 1, 10, 100, 1000 or 10 000, with *ρ* = 1 and *α* = 1. I also ran SFMs with ‘tuned’ values of *β* and *αρ* based on their estimated values in the ABMs (see below; with age effects: *β* = 0.85, *αρ* = 0.6; without age effects: *β* = 0.755, *αρ* = 0.975). For most models I used a default maximum survival probability *P* = 0.8. Separately, I tested effects of intra-population variation in *P* by running models with *P_i_* equalling each of 0.5, 0.55, 0.6, 0.65, 0.7, 0.75, 0.8, 0.85, 0.9 and 0.95. These models were run with the same values of *β*, *φ_ii_, ρ* and *α* as above. These values were chosen to represent a comprehensive range of conditions.

## 5. Agent-based models

Model outcomes can be affected by randomness and spatial arrangement of agents [24,25], particularly where models fail to capture spatially explicit [26], finite [27] or stochastic processes [28]. The SFMs described above, for example, assume that population sizes are infinite and are agnostic to the mode or spatial structure of competition. To evaluate how SFM predictions compare with finite systems with spatially explicit interactions, I tested mathematical model (SFM) predictions in an agent-based setting, using single-lattice agent-based cellular automata models (hereafter, ABMs). For simplicity, I imagined that a population of sessile organisms occupied a 100 × 100 cell lattice (10 000 cells total) with wrapping edges [29]. Each cell could be empty or contain one individual. I simulated genotypes of an arbitrary species, in which each genotype possessed a unique combination of 13 levels of tolerance (*T*) and 13 levels of suppression (*S*) abilities, each ranging from 0.2 to 0.8. All 169 genotypes were characterized by maximum survival probability *P*, maximum annual fecundity *F*, and age *A* in time steps, which were used to calculate age-dependent effects on *T*, *S* and *F*. Of note, unlike the mathematical SFMs, in the cellular ABMs terms A (kin assortment) and B (initiator effects), and their associated variables *α*, *β*, *ρ* and *φ_ii_* were not modelled explicitly. These could be measured, but took on emergent, dynamic values. Thus, ABMs served the dual purpose of (i) testing predictions of the SFMs in a stochastic, spatially explicit simulated community and (ii) showing what portions of the available parameter space simulated populations tended to occupy. ABMs do not simulate motile organisms and SFMs are not tested in that setting here. Details can be found in the electronic supplementary material.

## 6. Results

**Figure 2.**
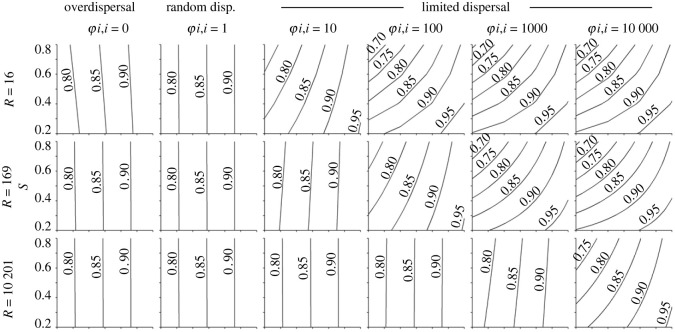
Contour plots of combined effects of *T* and *S* on fitness in SFMs, with tuned values of *αρ* = 0.96 and *β* = 0.85 for comparison with ABMs with age effects. Rows show models with different genotypic richness. Columns show models with different odds ratios of kin–kin encounters (*φ_i,i_*). The panel with *R* = 169 and *φ_i,i_* = 1 corresponds roughly to the ABM with random dispersal and age effects (figure 4, column 2), and the panel with *R* = 169 and *φ_i,i_* = 100 corresponds roughly to the ABM with limited dispersal and age effects (figure 4, column 4).

**Figure 3.**
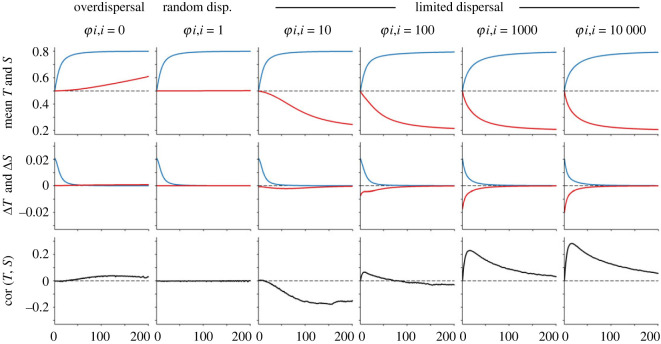
Temporal dynamics of SFMs with *R* = 169 and tuned values of *αρ* = 0.96 and *β* = 0.85 for comparison with ABMs with age effects. The first row plots these changes in mean *T* (blue, always upper) and *S* (red, always lower). The second row shows the changes in *S* and *T* from one time step to the next. The bottom row shows the correlation between *T* and *S* in each time step. Each column shows a model with different odds ratios of kin–kin encounters (*φ_i,i_*).

Tolerance was beneficial under all conditions (figures 2 and 3). This was expected, as survival varied linearly with *T_i_* (equation (3.6)) in the SFMs and ABM sub-models. By contrast, suppression, *S_i_*, had much more complex and situational fitness effects, as described below.

### 6.1. Neutral kin assortativity

**Figure 4.**
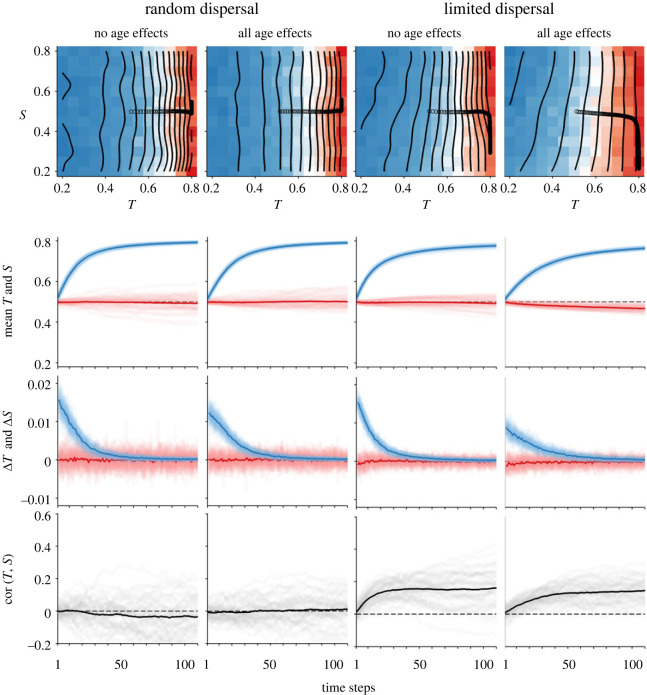
Temporal dynamics of selected ABMs. Heat maps in the top row show combined effects of *T* and *S* on fitness (Δ*N*; blue: negative, red: positive). The verticality of the contours indicates fitness depended only on *T*. Changes over time in population mean *T* and *S* are traced with dots. The second row plots these changes in mean *T* (blue, always top) and *S* (red, always bottom). The cloud of lines shows each of 50 replicates, with the mean of replicates in bold. Similarly, the third row shows changes in *S* and *T* from one time step to the next. The bottom row shows the correlation between *T* and *S* in each time step. Each column corresponds to a model with different dispersal modes and age effects.

In SFMs with neutral kin assortativity, meaning that genotype distributions were totally random, and with no initiator effects, meaning that individuals could not protect themselves from competition by killing neighbours, suppression had no effect on fitness (electronic supplementary material, figures S1 and S2) and cooperation was adaptively neutral. At maximum initiator effects, meaning that individuals that suppressed their neighbours experienced a complete, lifelong release from competition, suppression had a slight fitness benefit in the SFMs (electronic supplementary material, figures S3 and S4), weakly favouring defection. In 50 replicate ABMs with random dispersal there was no detectable benefit of neighbour suppression, despite initiator effects (figure 4). To determine whether fitness effects of *S* were exactly zero in the ABMs, or extremely close to zero, I ran *post hoc* models in which *S* varied, but not *T*, replicated 1000 times over 500 time steps. With all age effects enabled, suppression increased by approximately 1.1%, in 55.4% of simulations (exact binomial test, *p* = 0.0007; electronic supplementary material, figure S5) when *T* was 0.8, and by 7.4–11.6% in 68.7–87.2% of simulations (*p* < 0.0001) when *T* was 0.2. It was difficult to directly measure initiator effects, which varied dynamically, but *β* ranged roughly from 0.74 to 1.00. Parameters *φ_ii_*, *β*, *α* and *ρ* varied depending upon the model conditions and identity of surviving genotypes (electronic supplementary material, figure S6). Plugging in tuned estimates of *β*, *ρ* and *α* into SFMs essentially matched the outcomes of the ABMs.

*Positive kin assortativity.* When kin assortativity was positive, e.g. due to dispersal limitation or family aggregation, suppression was generally selected against (figures 2 and 3). With no initiator effects, neighbour suppression decreased fitness in all conditions (electronic supplementary material, figure S1). When initiator effects occurred, the initial selection favouring *S* was stronger as kin assortativity increased, and weaker as the number of genotypes decreased (electronic supplementary material, figure S3). However, selective benefits of neighbour suppression were short-lived, as they were tied to kin density (electronic supplementary material, figure S4). As with the neutral-association models, ABMs reflected the SFMs. Age effects did not alter this basic pattern (figure 3, electronic supplementary material, figure S7 and S8), although age effects on tolerance tended to exaggerate competitive disadvantages of neighbour suppression, and age effects on suppression tended to attenuate them. Agents in the dispersal-limited ABMs achieved a higher mean age than those in the random-dispersal ABMs, owing to the lower mean suppression in those models, although density was also higher, so the overall effect of both variables on population conditions were similar between dispersal types. The kin-assortativity odds ratio varied dynamically in the ABMs, peaking at about 250 : 1 and decreasing to 100 : 1 as equilibrium was reached, providing a reference point for interpreting *φ_ii_*: a value of 100–200 : 1 indicates extreme dispersal limitation (electronic supplementary material, figure S6).

Each genotype had a threshold density beyond which the fitness effect of suppression changed signs (see electronic supplementary material, methods for derivation)

6.1 $${\hat{s}}_{i}B_{i}=E(\hat{s}B)↔D_{i,\mathrm{threshold}}=\frac{\alpha \rho B_{i}\bar{S}-E(\hat{s}B)}{\left(\varphi_{ii}-1)(E(\hat{s}B)-\alpha \rho S_{i}B_{i}\right)}.$$

**Figure 5.**
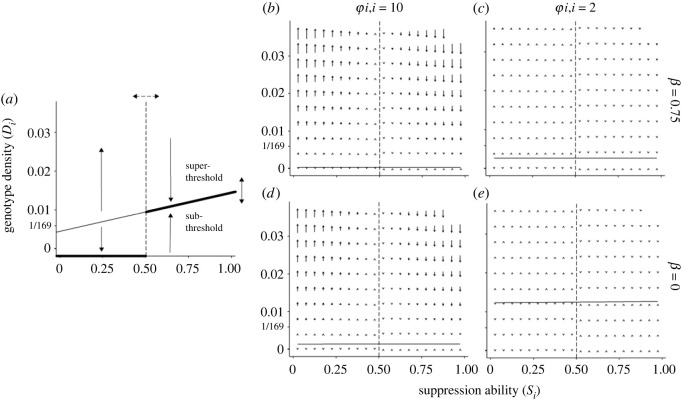
Effects of suppression ability and density on the direction of selection on fitness. The mean suppression of the community is shown with a dotted line (here, $\bar{S}=0.5$). Panel (*a*) shows an idealized example. If kin assortativity is positive, when weaker-than-average suppressors are introduced at a low density (lower-left quadrant), they will tend to decline, whereas stronger-than-average competitors will increase. The situation is reversed above the threshold density: weaker-than-average suppressors will increase and stronger-than-average suppressors will decline. Strong suppressors will tend to move toward the threshold density, and weak suppressors will be either extirpated or will experience runaway selection. Changes in the density of these individuals will affect the mean suppression of the community, which in turn will affect the threshold density. Examples are given which vary in the strength of kin assortativity and initiator effects (*b–e*). For these examples *p* = 0.9, *T* = 0.8, *αρ* = 0.9, *n* = 8.

Better-than-average suppressors benefitted when their density was low, and weaker-than-average suppressors benefitted when their density was high (figure 5). If weaker-than-average suppressors started above a threshold density, they would come to dominate via positive feedback. If they started below a threshold density, they would inevitably decline. By contrast, stronger-than-average suppressors declined above their threshold density, and increased below that density. The dynamics of the model depended upon whether it initialized in a super-threshold or sub-threshold condition for the weakest suppressors. The 169-genotype models tended to initialize in a super-threshold condition, in which case weak suppressors had a runaway fitness advantage when kin assortativity was positive. The 10 201-genotype models tended to initialize in a sub-threshold condition, in which case strong suppressors enjoyed a fitness advantage over weak suppressors, until the former reached self-limitation. In this metastable state, strong suppressors could oppress weak suppressors, which were never able to achieve a ‘critical mass’ density needed to switch the system to a super-threshold state. However, strong suppressors never came to dominate, due to self-limitation.

### 6.2. Negative kin assortativity

When kin assortativity was negative, e.g. due to over-dispersion, suppression conferred a fitness advantage under all conditions, although this advantage was weak relative to the advantages of tolerance. SFMs with no initiator effects and complete over-dispersal represent the best-case scenario for the evolution of neighbour suppression, in which case suppression was nonetheless orders-of-magnitude slower to evolve than tolerance (electronic supplementary material, figures S1–S4). ABMs behaved similarly to SFMs (figure 3, electronic supplementary material, figure S7 and S8), except that stochastic processes were amplified in the over-dispersal model, due to the fact that complete over-dispersal caused sudden and severe self-limitation in the spatial models (electronic supplementary material, figure S9).

### 6.3. Cooperation as an evolutionarily stable strategy

To be considered an ESS, a strategy must resist invasion by a small population using a different strategy. Defection, in the form of neighbour suppression, was an ESS in the cases when all genotypes were at a low, sub-threshold density and when kin assortativity was positive. Invasion by cooperators (i.e. weak suppressors) was impossible if those genotypes were introduced at low density. However, cooperators could invade if introduced at a density above their critical density, in which case the population would experience a state change and cooperators would come to dominate via positive-feedback mechanisms (electronic supplementary material, figures S10 and S11). Because the critical density was quite low, it is conceivable that stochastic processes in small populations could temporarily elevate cooperators above this density, causing change in the ESS, although this process was not specifically tested here. Conversely, if the population was in a super-threshold state with abundant cooperators, defectors could invade, but new cooperators could not. In this case, invading defectors would increase until reaching their threshold abundance. Seemingly, therefore, serial invasion by multiple suppressors could ‘poison’ the system by steadily weakening cooperators, but this was prevented by competition among defectors (electronic supplementary material, figure S10). In systems that initiated at a super-threshold state, cooperation dominated a stable mixed-strategy community with defectors stably persisting at low abundance.

### 6.4. Model generality and conditionality

Tolerance always provided a fitness benefit. Fitness effects of neighbour suppression, however, varied in direction and magnitude according to the value of every variable in the model. Cooperation was an ESS whenever there was positive kin assortativity and cooperator densities were high enough or initiator effects were low enough that cooperators were above their threshold densities. Otherwise, defection would be a meta-stable ESS when kin assortativity was positive, a stable ESS when kin assortativity was negative, or a stable ESS when kin assortativity was neutral and initiator effects were present (electronic supplementary material, figure S12). Thus, throughout most of the parameter space explored in this study, cooperation was an ESS, albeit dependent upon both current and initial conditions.

**Figure 6.**
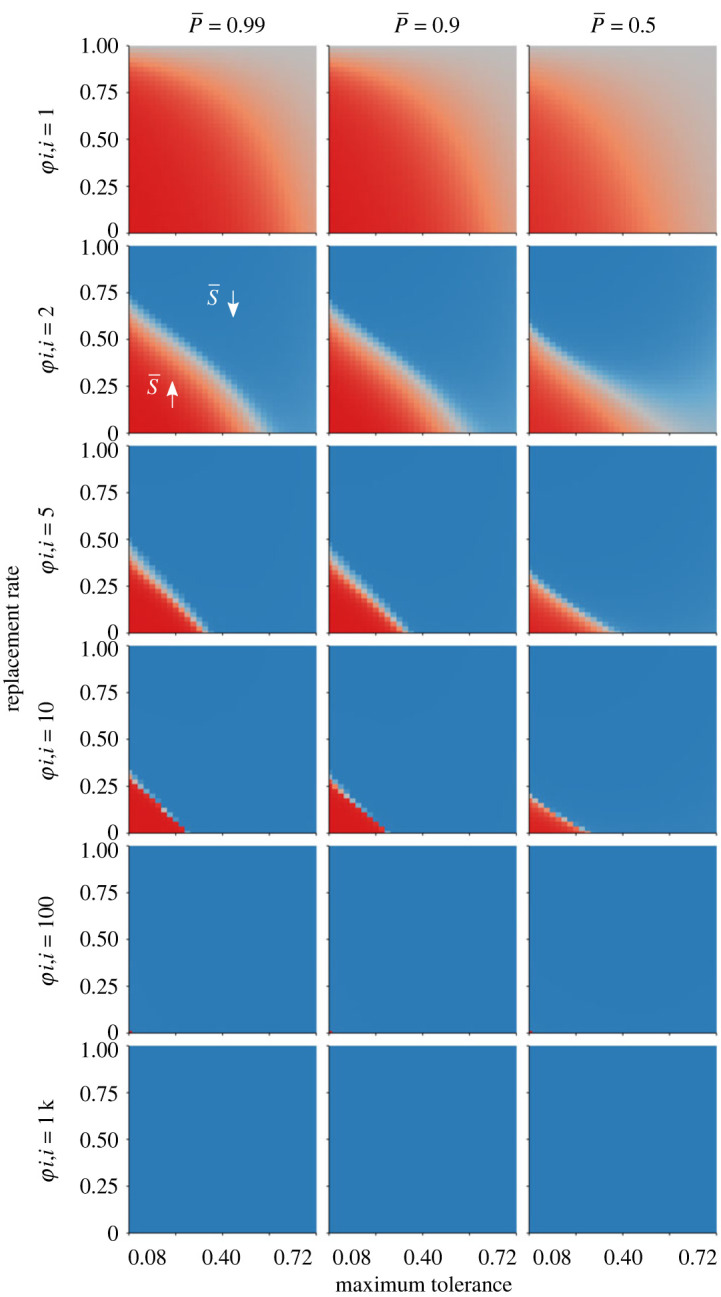
Effects of maximum tolerance ability (i.e. the overall strength of competition) and the replacement rate (*β*) on the dominant ESS (red = defection favoured, blue = cooperation favoured). Panels show models varying in average survival probability and the strength of kin assortativity. Suppression was favoured only when kin assortativity was low, replacement rates were low, and competition was very intense (maximum *T* less than about 0.3, such that competition caused greater than or equal to 70% reductions in survival probability). For these models *R* = 169, *n* = 8, and *αρ* = 1.

In the case of positive kin assortativity, the key consideration was the threshold density, which in turn was influenced by the mean strength of the suppression field ($\bar{S}$), degree of kin assortativity (*φ_ii_*), number of interactors (*n*), age effects (*α*), population density (*ρ*), neighbour replacement rate (*β*), suppression ability (*S_i_*) and mean population tolerance ($\bar{T}$). Most of these variables exerted their influence through the initiator effects term, B, attenuating their effects on fitness (as B itself had weak effects on fitness). The exception was the mean population tolerance, $\bar{T}$, which had a negative linear relationship to B and which exerted large effects on evolutionary outcomes. Generally, tolerance was strongly favoured, and so $\bar{T}$ rose quickly to the maximum allowed value of *T_i_*. In most models reported here, that value was set to be 0.8—which resulted in initiator effects being diminished by 80%. However, limiting *T_i_* to a lower maximum value would consequently strengthen initiator effects. Stronger initiator effects would raise the density-threshold for the evolution of defection, in turn expanding the number of conditions in which neighbour suppression could evolve (figure 6). As a result, the equilibrium mean tolerance $\bar{T}$ had outsized effects on evolutionary outcomes. In plain terms, in systems where competition harshly reduces fitness (as compared with the competition-free condition), suppression will more likely be favoured. In systems where competitive effects are milder, de-escalation will more easily evolve.

The importance of tolerance versus suppression as determinants of fitness (measured as the partial derivative of fitness with respect to tolerance and suppression, see electronic supplementary material, methods) depended strongly on the number of interaction partners, *n*, with *n* = 1 representing pairwise competition. In general, suppression was more likely to emerge as an ESS as *n* decreased to one. It has been suggested that in the pairwise mode tolerance and suppression should contribute equally to fitness, meaning that competitive escalation should be quick to evolve in pairwise competition [14,30,31], but this was not quite the case in these models. Instead, suppression was rarely more than half as important as tolerance at determining fitness, except in the case of very high population densities, weak age effects, extremely strong competition and neighbour replacement rates effectively equalling zero. More generally, fitness effects of suppression tended to be 60–90% weaker than fitness effects of tolerance in the pairwise case (*n* = 1), and much less in the multiplayer case (*n* > 1; electronic supplementary material, figure S11). The exceptions were systems with very high kin assortativity, in which de-escalation evolved at a rate up to approximately 33% the rate of evolution of increased tolerance. Thus, regardless of whether selection favoured competitive escalation or de-escalation, it tended to act slowly relative to selection favouring increased competitive tolerance.

In the SDMs used in this study, competition occurred in a two-dimensional community of sessile organisms, at high density (i.e. similar to competition among plants). Kin assortativity was manipulated by varying the mode of dispersal. In the SFMs, competition was not spatially explicit and the models themselves are agnostic to both the mechanism of competition and the ecological process influencing kin assortativity. Thus, while the SDMs most closely represent sessile competitors, the SFMs are generalizable to a wide variety of systems, as governed by a variety of assumptions, including but not limited to: competition results in a loss of survival probability; competitor encounter rates are linearly related to competitor density; a bias towards or against encountering kin affects all kin (and all unrelated individuals) equally; there is no kin recognition; competitor replacement has a constant chance of happening; and tolerance of competition is possible and is uncorrelated with suppression ability.

## 7. Discussion

Does an individual benefit by harming direct competitors? For decades, we thought that the answer is ‘yes’, because competitor suppression provides greater access to resources and, presumably, a lower likelihood of being competitively inhibited via the ‘initiator effect’ [30,32,33]. Thus, in the absence of trade-offs, it has been assumed that competition should always push organisms towards conflict. In this study, game theoretic models showed that, while suppression may produce neighbourhood-scale benefits via the initiator effect (e.g. equation (2.1)), these were vanishingly weak and easily overridden by a risk of self-inhibition so severe that in many modelled conditions suppression of neighbours conferred a strong fitness *cost*. Thus, altruism, in the form of competitive de-escalation, may be an evolutionarily stable strategy (ESS) in a wide range of systems, and, crucially, in the complete absence of any costs of competition.

One of the key outcomes of this study is support for the hypothesis that diffuse interactions may dominate multiplayer competition in a wide variety of conditions, dramatically reducing the importance of individual pairwise interactions [13,15–17,19]. While not all individuals experience the same competitive neighbourhood, evolution and population growth concern the population overall, as statistical sampling diffuses effects of individual interactions [15,17]. Further amplifying diffuse effects is the fact that each individual interacts with many neighbours, and the effects of individual competitors are diluted as they are distributed among these neighbours [15,16]. In the idealized special case in which all interactions are diffuse (e.g. *φ*_ii_ *=* 1 and *B* = 1; electronic supplementary material, figures S1 and S2), competition functioned as a uniform field, affecting all genotypes more or less equally. A useful analogy is an electromagnetic field, which is created by, and also directs the motions of, individual particles within the field. Here, individual competitors contribute to the ‘suppression field’, which applies evenly to all others in the community. These ‘suppression-field’ models suggest that in multiplayer competition diffuse interactions are likely to be far and away the dominant mode of competition. If this is true, we may be thinking of competition in the wrong way.

It is tempting to view competition as fundamentally a pairwise interaction, and multiplayer competition as an upscaled network of such pairwise interactions. While this is certainly true, using this pair-centric mode of thinking it is difficult to intuit how diffuse interactions manifest or what their role is in evolutionary dynamics of competitor communities. I propose instead that we should take a different perspective and consider using a mean-field, fully diffuse model as our starting point for thinking about the evolution of competitive ability in realistic communities of multiple individuals. In other words, we should begin with the premise that individual interactions do not matter, because they are completely diluted and diffused by Type I and III diffuse effects. The question then becomes, what processes cause a community *not* to function this way, and instead cause individual interactions to become important? This is a much simpler, more straightforward way of thinking about multiplayer competition than an upscaled pair-centric paradigm. In these models, two processes disturbed the competition mean field, enabling suppression to evolve as a strategy: (i) kin assortativity and (ii) the initiator effect. Positive kin assortativity—i.e. caused by dispersal limitation or communal behaviour—caused a positive correlation between the suppression caused and the suppression felt by a genotype. Kin assortativity burdened competitors with a risk: by suppressing their neighbours they might inadvertently harm their own relatives. Conversely, weak suppressors benefitted by assorting together; in so doing they protected themselves from the strong competition experienced by other community members. I note that these models incorporate inclusive fitness organically without the need for it to be specified explicitly.

Whether competitive escalation or de-escalation was an ESS depended upon a variety of conditions, such as the strength of age effects, the population density, the mean strength of the suppression field, the mean tolerance ability of neighbours, the size of the competitor neighbourhood (i.e. the number of competitors an individual encounters), the odds of kin–kin encounter, and the replacement rate of killed competitors. Although these effects are mathematically complex, the ecological interpretations are intuitive (table 1). Any condition that made competition stronger increased the value of neighbour suppression, which made the initiator effect stronger. For example, in a community where competition is intense, strongly size-asymmetric, long-lasting, and/or subject to strong priority effects, suppression is likely to be favoured. Conversely, in systems where competition is relatively weak and/or dispersal limitation is important, cooperation is more likely to be favoured. However, if neighbour recognition allows targeted responses to related versus unrelated neighbours—which is not accounted for in the present study—suppression of unrelated neighbours could evolve. On the other hand, high niche overlap among kin could produce the opposite effect, making suppression costly. The key consideration is the correlation between the competitive environment of an individual (${\hat{s}}_{i}$) and its competitive ability (*S_i_*). If stronger suppressors find themselves also experiencing stronger competition, then cooperation will evolve. Where do typical ecological systems fall along this spectrum? Data from the field are needed to answer this question, but the models point towards some intuitable hypotheses (electronic supplementary material, table S1). I note that even in the best-case scenario suppression was orders of magnitude slower to evolve than tolerance in models. If the same holds true for ecological systems, then fitness benefits of suppression might be difficult to detect or swamped by other ecological or evolutionary processes.

Models suggest that the evolutionary outcome of competition also depends strongly on the relative abundance of suppressor genotypes. This could matter, for example, during species invasions. When a species is at low abundance, suppression is favoured because kin–kin encounters are rare. However, as the species rises in abundance due to competitive exclusion, kin–kin encounters become more common, and the system might eventually change state to one where cooperation is favoured. Thus, our models point to a novel eco-evolutionary hypothesis for the evolution of competitiveness in invasive species: based purely on competition, the suppression-field model predicts that competitive aggression will be favoured in the early stages of invasion but will give way to cooperation once the species has established.

Mean-field models are finding expanding use in disciplines concerned with complex systems with large numbers of agents, such as economics, social science, physics and neurobiology [23]. Recent advances have begun to link mean-field models with game theory [34], allowing researchers to explore strategic optimization and equilibration of complex systems, including competition. Applications include optimizing competitive harvest of forests [35], evolution of strategic alliances in competing organisms [36], economic market competition [37] and more [38]. Mean-field game theory has enormous potential to help us conceptualize competition in natural communities and drive hypothesis formation that addresses the messy and complicated nature of multiplayer competition in ways that are not achievable with pairwise studies.

The field models developed here point towards a new way of conceptualizing multiplayer competition as a predominantly diffuse field that is occasionally perturbed by individual-based interactions. From that perspective, a question emerges: what processes cause individual-based interactions to become important? I tested kin assortativity and competitor replacement lag as two candidate processes, but many others certainly exist. For example, my models did not account for kin recognition, quorum sensing or learning, all of which might influence the evolution of cooperation. Furthermore, the direction of selection on cooperation versus defection was complex and contingent on many processes, such as priority effects, age effects and genotype density. Other, unmodelled processes are also likely to be important, such as higher trophic interactions, ecological trade-offs, modes of inheritance, motion of competitors, etc. Much more work is needed both to generalize these findings and to guide the development of ecological experiments to test the hypotheses generated by these models.

## Data Availability

Data access is available on Dryad (https://doi.org/10.5061/dryad.000000075) [39]. The data are provided in electronic supplementary material [40].
